# Engineered exosomes: a promising approach for overcoming challenges in pancreatic cancer therapy

**DOI:** 10.1186/s12951-025-03697-0

**Published:** 2025-09-29

**Authors:** Mo Sha, Yang Gao, Xu Yin, Xueyao Li, Caiqi Liu, Shuang Li

**Affiliations:** 1 Department of Hepatobiliary and Pancreatic Surgery, The Second Hospital of Jilin University, Jilin University, Changchun, 130000 P. R. China; 2 Department of Gastrointestinal Medical Oncology, Harbin Medical University Cancer Hospital, Harbin, 150081 P. R. China; 3 Heilongjiang Province Key Laboratory of Molecular Oncology, Harbin, 150081 Heilongjiang Province P. R. China

**Keywords:** Engineered exosomes, Pancreatic cancer, Delivery system, Cancer therapy

## Abstract

Pancreatic cancer (PC) is among the deadliest types of cancer, with very low chances of survival. It is often asymptomatic in the early stage, making diagnosis difficult. Therefore, it is typically found at an advanced stage, resulting in patients missing the opportunity for radical surgery. The complex biological characteristics of PC, coupled with the difficulties in drug delivery and tumor resistance, limit the effectiveness of drug therapy. Due to their compatibility with biological systems and low likelihood of triggering an immune response, exosomes are seen as a promising method for drug delivery. They are capable of targeting and penetrating tissues inside the body and can be engineered through surface modification and drug loading. Engineered exosomes possess controllable and diverse drug-carrying capabilities, which can enhance drug internalization and cellular uptake. Owing to their special properties and the potential to overcome the drawbacks of standard therapies, engineered exosomes have appeared as a promising treatment option. This review aims to comprehensively summarize the current application status and progress of engineered exosomes used in PC therapy.

**Graphical abstract:**

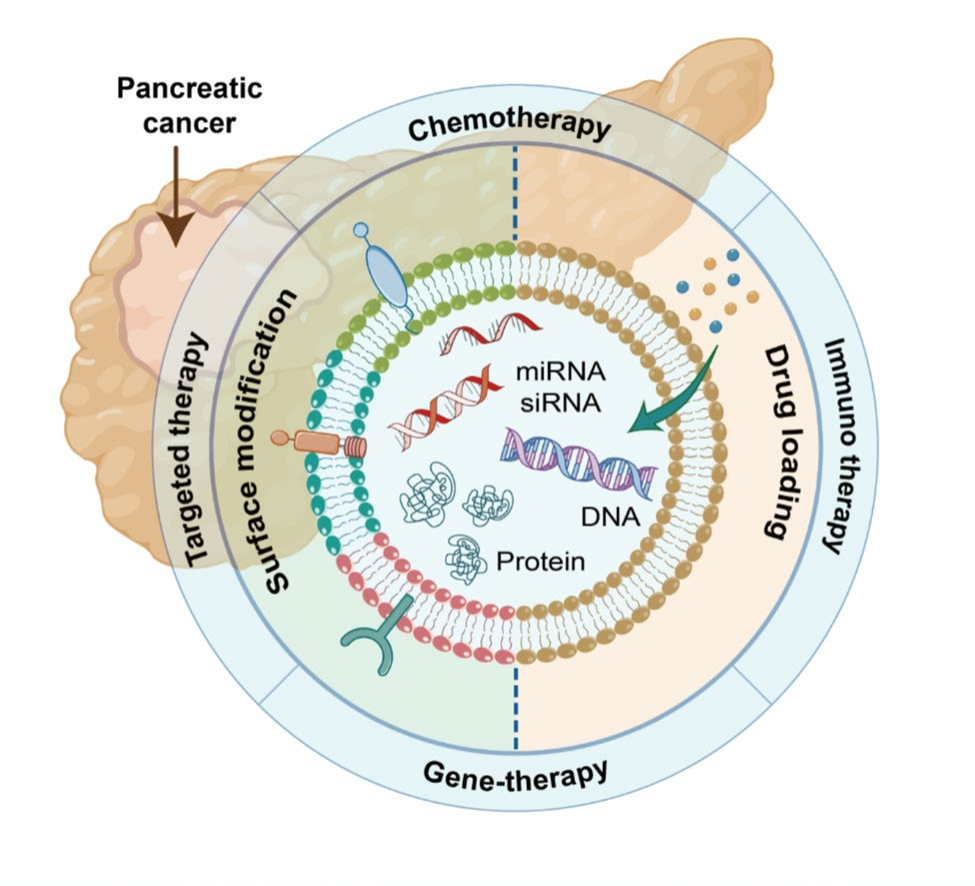

## Introduction

Pancreatic cancer (PC) is an extremely aggressive type of malignant tumor, known for its high death rate and brief survival times [1–3]. Early detection remains a formidable challenge, as PC is typically asymptomatic in its initial stages. The most widely used serum biomarker, carbohydrate antigen 19 − 9 (CA19-9), suffers from limited specificity and a high false-positive rate [4]. Likewise, conventional imaging modalities such as enhanced computed tomography (CT), magnetic resonance imaging (MRI), and endoscopic ultrasound (EUS) demonstrate inadequate sensitivity for identifying early-stage lesions [5]. These limitations collectively account for the difficulty of early diagnosis, resulting in the majority of patients being diagnosed only at advanced stages, when the disease has frequently metastasized and the best window for curative surgery has been missed [6]. Although surgical excision remains the only potentially curative treatment, its benefits are substantially restricted by delayed diagnosis, leaving most patients unable to achieve long-term survival [7].

The complex biological characteristics of PC further exacerbate the difficulty of treatment. Cancer cells exhibit high heterogeneity and invasiveness, enabling them to rapidly penetrate surrounding tissues and blood vessels, leading to rapid tumor spread. Beyond late diagnosis, the highly complex and immunosuppressive tumor microenvironment (TME) further limits therapeutic efficacy. The TME in PC is highly intricate and strongly immunosuppressive, enriched with extracellular matrix (ECM), regulatory T cells (Tregs), myeloid-derived suppressor cells (MDSCs), and tumor-associated macrophages (TAMs), along with elevated levels of inhibitory cytokines and immune checkpoint molecules [8]. Together, these components create a formidable immunosuppressive barrier that not only hinders the cytotoxic activity of immune cells but also reduces the effectiveness of anti-tumor drugs [9–11].

In terms of drug treatment, PC also faces numerous challenges. Although traditional chemotherapy drugs can inhibit tumor cell proliferation to a certain extent, their effectiveness is limited, and they are often accompanied by severe side-effects. In recent times, the advancement of new targeted and immunotherapy medications has been ongoing, yet their therapeutic impact is constrained by challenges in drug tolerance and delivery. Drugs struggle to effectively penetrate the barriers within the TME, resulting in insufficient delivery to tumor cells and poor treatment outcomes [12].

Against this backdrop, exosomes, as a novel drug delivery system, have attracted extensive attention [13, 14]. Derived from diverse cellular sources including mesenchymal stem cells, immune cells, and tumor cells [15], exosomes exhibit excellent biocompatibility and low immunogenicity [16]. These unique properties allow them to circulate widely within the body while minimizing immune rejection and inflammatory responses to foreign substances, thereby enhancing the safety and tolerability of drug delivery [17]. The surface of exosomes is enriched with molecules involved in cell recognition and signal transduction, enabling specific binding to target cell receptors and thus achieving precise drug delivery [18, 19].

In addition, exosomes possess the remarkable capability to traverse multiple biological barriers, including the blood–pancreas barrier, the dense stromal matrix of the TME, and even the blood–brain barrier [20]. This ability directly addresses one of the major obstacles in PC treatment—inefficient intratumoral drug delivery—thereby presenting new opportunities for managing this difficult-to-treat disease [21]. Furthermore, exosomes can be engineered through genetic or chemical modifications to enhance their therapeutic efficacy. For instance, specific targeting ligands such as antibodies or peptides can be introduced onto the exosome surface to improve tumor specificity [22]. In addition, fluorescent markers or magnetic nanoparticles can be incorporated for real-time tracking and imaging. Drug loading methods are also diverse: beyond encapsulating drugs within exosomes, active agents can be immobilized on the exosomal membrane through chemical conjugation or physical adsorption, further improving loading efficiency and stability [23].

Engineered exosomes possess controllable and diverse drug-carrying capabilities, enabling them to load various types of drugs according to therapeutic needs, including small molecule drugs, protein-based drugs, and nucleic acid-based drugs [24]. The lipid bilayer structure of exosomes protects drugs from degradation by endogenous enzymes and harsh physiological conditions, thereby maintaining drug stability, prolonging their half-life, and preserving therapeutic activity. Upon fusion with target cells, exosomes directly release their payload into the cytoplasm, bypassing extracellular degradation and ensuring efficient internalization, which further promotes drug uptake and enhances anti-tumor activity [25].

In summary, the difficulty of early diagnosis, the immunosuppressive TME, and formidable barriers to drug delivery underscore the urgent need for innovative therapeutic strategies in PC. Engineered exosomes, with their unique physical and biological properties, broad cellular origins, and superior ability to traverse biological barriers, represent a transformative therapeutic platform capable of overcoming the limitations of conventional therapies and significantly improving patient prognosis [26, 27]. In this review, we provide a comprehensive overview of the current challenges in PC drug therapy and summarize the application status and latest advances in engineered exosome-based treatments, with a particular focus on chemotherapy, immunotherapy, gene therapy, and targeted therapy. We also highlight their potential in enhancing drug efficacy, overcoming resistance, and improving clinical outcomes, offering novel insights and strategies for the future management of PC.

## Problems in pancreatic cancer drug therapy

### Tumor biological characteristics

Tumors display profound intratumoral heterogeneity, with different cancer cells in the same PC lesion showing significant differences in gene expression, metabolic characteristics, and drug sensitivity [28]. This intratumoral heterogeneity means that even if a certain drug is effective against some cells in the tumor, it may be ineffective against others, resulting in poor treatment outcomes [29]. For instance, genetic mutations may enable subsets of tumor cells to acquire resistance to targeted drugs, allowing these resistant cells to survive and continue proliferating during treatment, ultimately driving tumor recurrence and progression. Such drug resistance not only limits the efficacy of monotherapies but also complicates and introduces uncertainty into combination treatment regimens [30].

In addition to cellular heterogeneity, TME plays a pivotal role in PC progression and therapeutic resistance. Tumor-associated macrophages (TAMs) and cancer-associated fibroblasts (CAFs) are central effectors in shaping this immunosuppressive milieu. TAMs, predominantly of the M2 phenotype, secrete immunosuppressive cytokines and metabolic products such as arginase and reactive oxygen species (ROS), directly inhibiting T-cell activity [31], while simultaneously recruiting regulatory T cells (Tregs) to reinforce the immunosuppressive network [32]. CAFs contribute by secreting dense ECM components that act as physical barriers preventing CD8 + T-cell infiltration [33]. Moreover, CAFs release soluble factors including stromal cell-derived factor 1 (SDF-1) and interleukin-6 (IL-6), which polarize TAMs toward the M2 phenotype [34] and promote Treg proliferation [35]. The interplay of TAMs and CAFs thus amplifies immune suppression and reinforces drug resistance, significantly reducing therapeutic efficacy.

Beyond intratumoral variability, inter-patient heterogeneity further complicates PC management. Differences among patients in tumor origin, developmental trajectory, and TME composition present major obstacles to the design of universally effective treatment regimens [36, 37]. Therefore, while personalized treatment is urgently needed, achieving it remains a formidable challenge. These differences arise not only from intrinsic tumor cell characteristics but also from patient-specific factors, the complexity of the TME, and dynamic alterations during therapy. As a result, developing reliable biomarkers capable of predicting therapeutic responses and guiding personalized treatment strategies has become an important focus of contemporary PC research [38].

Finally, PC employs diverse immune evasion mechanisms, further hindering the success of drug therapy. Tumor cells evade immune surveillance by downregulating surface antigen expression, secreting immunosuppressive factors such as transforming growth factor-β (TGF-β) and interleukin-10 (IL-10), and recruiting suppressive immune populations such as Tregs and MDSCs [39]. CAFs and TAMs synergistically enhance these effects through ECM deposition and immune suppression, together contributing to the limited efficacy of immune checkpoint inhibitors [8]. For instance, even with checkpoint blockade, immune recognition of tumor cells may remain insufficient, preventing effective antitumor immune responses [40, 41].

### Drug delivery challenges

The ECM barrier of PC cells leads to low drug delivery efficiency. The TME of PC is abundant in ECM components like collagen and fibronectin, creating a thick matrix barrier [42, 43]. CAFs are major contributors to this barrier, actively secreting ECM components including collagen, fibronectin, and hyaluronan. This excessive ECM deposition increases stromal stiffness and interstitial fluid pressure, further limiting drug diffusion and distribution [44]. In addition, CAF-driven matrix remodeling facilitates the recruitment and polarization of TAMs, neutrophils, and MDSCs, thereby amplifying immune suppression and reducing intratumoral drug permeability. As a result, even when drugs succeed in entering tumor tissue, their intratumoral distribution is often uneven, leaving subsets of tumor cells insufficiently exposed to therapeutic concentrations [45].

Moreover, structural and functional abnormalities of tumor vasculature in PC further exacerbate these challenges. Tumor blood vessels typically exhibit poor permeability and inadequate perfusion [46, 47]. These defects not only restrict the overall amount of drugs reaching the tumor but also lead to abnormal drug retention and heterogeneous distribution within the tissue. For example, macromolecular therapeutics or nanodrugs often fail to penetrate the aberrant vascular walls, limiting their ability to reach tumor cells and exert therapeutic effects [48].

Additionally, drug metabolism and excretion represent another major obstacle. The pancreas, characterized by its abundant blood supply, has a strong metabolic capacity [49]. Consequently, drugs may undergo rapid metabolism and clearance in the liver and kidneys, reducing systemic circulation time and lowering effective drug concentrations at the tumor site. Furthermore, interactions between some drugs and pancreatic digestive enzymes can compromise their stability and biological activity, further diminishing treatment efficacy [50].

### Drug resistance issues

Some PC patients exhibit primary resistance, indicating that their tumor cells do not respond to drug treatment from the outset. This resistance may be linked to intrinsic gene mutations within the tumor cells or to the abnormal activation of signaling pathways [51]. For example, KRAS mutations—present in up to 95% of pancreatic ductal adenocarcinoma (PDAC)—drive continuous activation of MEK and PI3K/AKT signaling pathways, mediating chemoresistance [52]. Mutant KRAS also upregulates CD47 to inhibit macrophage-mediated phagocytosis, promoting immune evasion [53]. In addition, dysregulation of the Cyclin D–CDK4/6 pathway, particularly CDK6 overactivation, phosphorylates RB protein and enhances CDK2 activity, thereby bypassing G1/S checkpoints, driving angiogenesis, tumor stemness, and immune suppression [54, 55].

In addition, there is also acquired resistance within PC patients. Even if the initial treatment is effective, tumor cells may develop resistance over time [56]. Under the selective pressure of drug treatment, tumor cells can adapt and develop resistance to the effects of drugs through gene mutations, phenotypic changes, and the activation of alternative signaling pathways. A critical phenotypic mechanism is epithelial-mesenchymal transition (EMT), during which epithelial cells lose polarity and acquire mesenchymal features, increasing invasiveness and drug resistance [57]. EMT also promotes immune resistance by upregulating checkpoint proteins such as PD-L1 and CTLA-4, activating autophagy to evade cytotoxic T lymphocyte (CTL)-mediated lysis, and impairing IFNγ signaling and antigen presentation[58]. For example, tumor cells may also increase the expression of drug efflux pumps to expel drugs from the cells, or activate alternative pathways to circumvent drug targets and continue to promote cell proliferation and survival. Ultimately, this leads to a reduction in drug efficacy or complete treatment failure [59].

### Evaluation and monitoring challenges

In clinical settings, there is an absence of sensitive indicators for evaluating efficacy. Currently, the commonly used efficacy evaluation criteria, such as the RECIST criteria, mainly rely on changes in tumor size to judge treatment outcomes. However, for PC, which is highly invasive and prone to micrometastasis, relying solely on changes in tumor size is insufficient to accurately reflect the true efficacy of drug treatment [60]. Moreover, some novel drugs may exert their therapeutic effects by altering the biological behavior of tumors or inhibiting tumor angiogenesis, which may not lead to significant changes in tumor size in the short term. This may result in an underestimation of the treatment effect [61].

Furthermore, during the drug treatment process, there is a lack of effective real-time monitoring methods. Understanding how tumor cells react to drugs and develop resistance in a timely fashion is not feasible [62]. Current imaging examinations and blood biomarker tests often have specific limitations and delays, and cannot offer clinicians timely and accurate bases for treatment adjustments [63]. For example, imaging examinations usually require a certain time interval to observe tumor changes [64], the specificity and sensitivity of blood biomarkers are suboptimal, making it challenging to accurately represent the current status of tumors [65].

Emerging approaches such as liquid biopsy and molecular imaging show great promise for real-time monitoring. Liquid biopsy enables non-invasive detection of circulating tumor cells (CTCs), circulating tumor DNA (ctDNA), and tumor-derived exosomes, providing dynamic molecular profiles of tumor burden, clonal evolution, and treatment response [34]. Meanwhile, advanced molecular imaging techniques such as PET/CT allow spatial localization of lesions and visualization of specific molecular activities within the tumor, complementing liquid biopsy. Together, these methods may overcome the limitations of conventional monitoring and facilitate personalized treatment adjustments in PC.

**Fig. 1:**
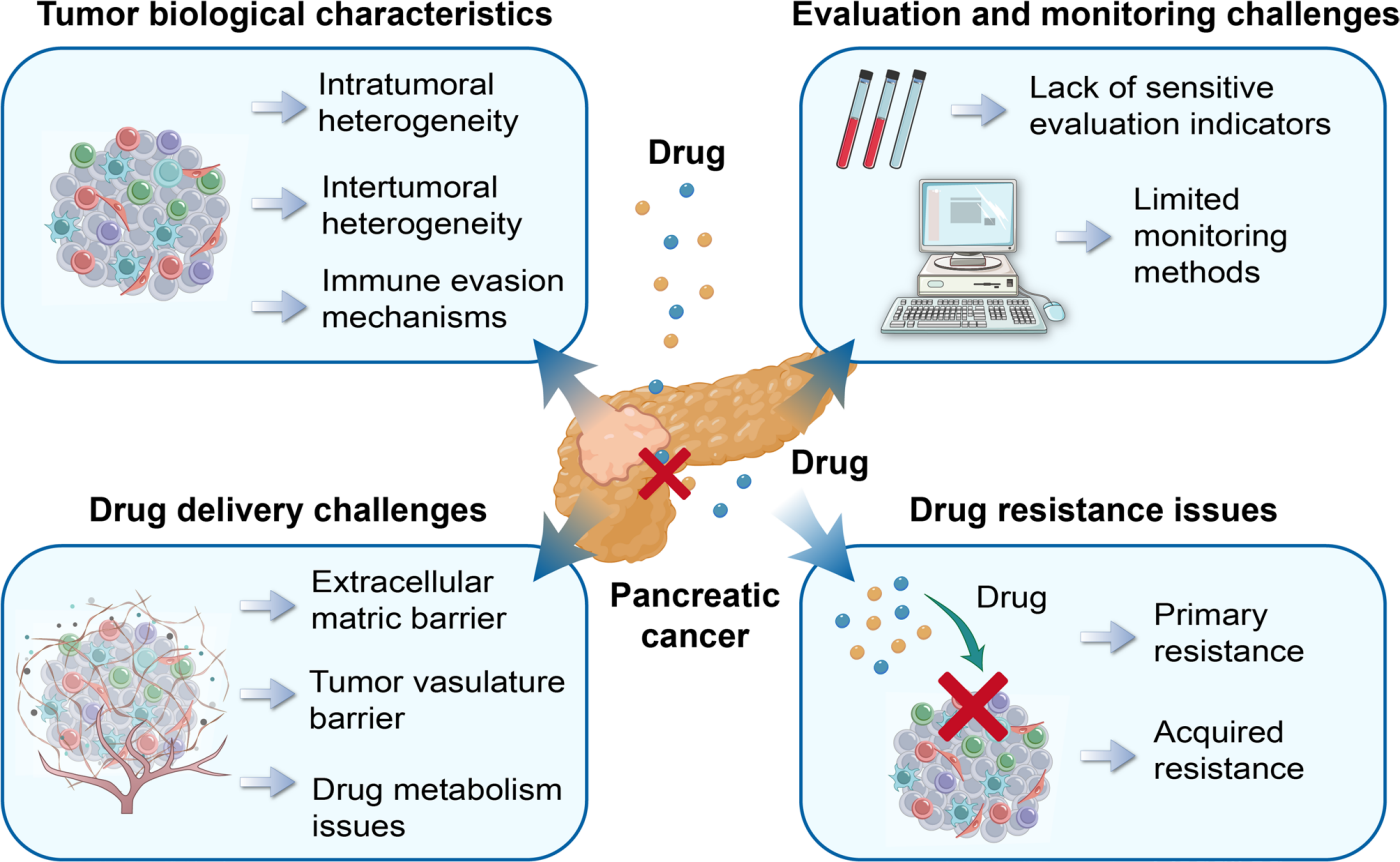
Problems in PC drug therapy. This figure details the challenges faced in PC drug therapy, covering tumor biological characteristics, drug delivery challenges, drug resistance issues, and evaluation and monitoring challenges

As shown in Fig. 1, we summarize the challenges faced in PC drug therapy, covering tumor biological characteristics, drug delivery challenges, drug resistance issues, and evaluation and monitoring challenges.

## Overview of engineered exosomes

### Advantages of exosomes as drug carriers

**Fig. 2:**
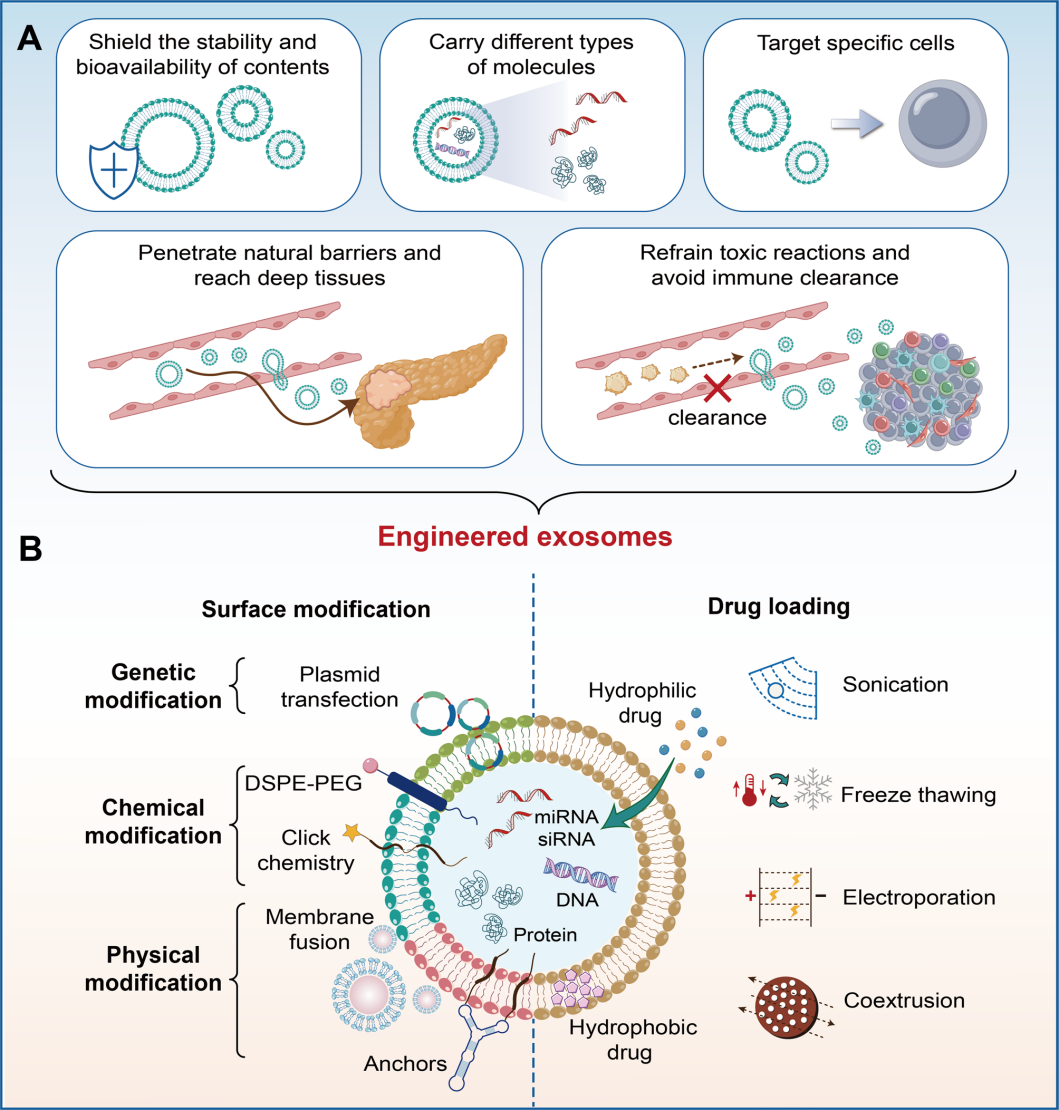
Preparation and advantages of engineered exosomes. (**A**) The advantages of engineered exosomes in vivo. (**B**) The preparation methods of engineered exosomes, including surface modification and drug loading techniques

Exosomes, which are nanoscale vesicles with membranes, are secreted by cells and usually measure between 30 and 200 nanometers [66]. The natural targeting ability of exosomes alone is insufficient to ensure their recruitment to specific sites; therefore, modifications are necessary to enhance their targeting precision and enable more accurate delivery [67]. These vesicles are capable of carrying bioactive molecules like microRNAs and proteins, facilitating communication between cells. Their therapeutic potential in tumor treatment has been widely validated in numerous studies [68]. However, unmodified natural exosomes have limited targeting capabilities. Once in the body, exosome-based drugs are often rapidly cleared by the circulating immune system or absorbed by non-target cells, resulting in a shortened drug half-life. Macrophages internalize exosomes through an energy-dependent, actin- and PI3K-mediated phagocytosis process, sorting them into phagosomes for ultimate lysosomal degradation [69]. Furthermore, there is compelling evidence that the CD47–SIRPα “don’t eat me” checkpoint applies to exosomes, enabling them to evade phagocytosis and prolong their circulation. CD47 is widely expressed on exosomes from various cell types and binds to SIRPα on macrophages and monocytes, inhibiting phagocytosis through the same myeloid immune checkpoint used by these cells [69]. Exosomes can be engineered through genetic or chemical modifications to enhance particular functions or gain new capabilities (Fig. 2A) [24]. The potential applications of exosomes in drug delivery, disease diagnosis, and therapeutic intervention can be enhanced by modifications like surface decoration, drug loading, and the introduction of targeting ligands [26].

### Exosome surface modification

Surface modification of exosomes not only endows them with targeting abilities but also enables in vivo imaging, tracking, and interaction with other biological systems by introducing functional molecules(Fig. 2B). This provides new tools for personalized and precision medicine. The main methods of exosome surface modification are divided into chemical modification, genetic engineering modification, and physical modification [70]. Chemical modification of the exosome surface offers flexibility, allowing for the binding of diverse ligands and achieving more efficient and biocompatible binding. However, this carries the risk of altering native surface proteins or compromising membrane integrity. Genetic modification can maintain the stability of membrane proteins or peptides and is well-suited for applications requiring precise, long-term expression of membrane-anchored targeting moieties. Physical modification, on the other hand, allows for remote control of targeting and payload release and is particularly suitable for applications where external triggers (magnets, ultrasound, light) are used to activate release or localization [71]. In oncology applications, RGE or GE11 peptides have been chemically linked to exosomes to target gliomas or EGFR-positive breast cancer, effectively delivering siRNA or chemotherapeutic drugs. HEK293 exosomes expressing Lamp2b-iRGD and loaded with doxorubicin targeted αv integrin-positive tumors and inhibited growth in a breast cancer model [72]. Neutrophil-derived exosomes modified with SPIONs, coupled to a magnetic field, aggregated and delivered TNF-α at the tumor site, reducing toxicity and improving targeting [15].Among them, chemically, click chemistry enables target molecule conjugation via specific reactions, PEGylation can prolong circulation time, but most people develop anti-PEG IgM or IgG antibodies [73]. These antibodies can affect the behavior and tolerability of the nanocomplex [74]. Receptor-ligand interactions facilitate modification through biomolecular specificity, hydrophobic insertion embeds hydrophobic molecules directly into exosome membranes through electrostatic interactions leveraging charge properties for molecule attachment. Genetically, plasmid DNA or mRNA with target gene sequences is introduced into parental cells, resulting in the natural expression of corresponding targeting molecules on the exosomes they generate [75]. Physically, membrane fusion introduces new molecules by merging exosome membranes with others under altered conditions, while anchoring fixes modifier molecules using hydrophobic or lipophilic substances. Collectively, these methods offer versatile options for tailored exosome surface modification.

### Exosome functional loading

As drug delivery carriers, exosomes can deliver hydrophilic, hydrophobic, and amphiphilic substances. Currently, based on different engineering targets, exosome loading methods can be categorized into endogenous and exogenous loading [76]. Endogenous loading is an engineering-based method that relies on parental cells to incorporate particular therapeutic molecules, such as microRNAs and proteins, into the natural components of exosomes. Initially, the exosome donor cells are engineered. Drugs are introduced into these donor cells using methods such as transfection and co-culture. Subsequently, the donor cells load the drugs into the exosomes they secrete, resulting in engineered exosomes that contain the drugs. Endogenous loading involves first introducing specific therapeutic molecules (such as microRNAs and proteins) into cells through gene transfection, followed by selective packaging via exosome sorting, collection of cell culture supernatant, and isolation of vesicles using ultracentrifugation, size exclusion chromatography, or similar purification methods to collect exosomes loaded with the specific therapeutic molecule. Finally, endogenous loading efficiency is verified by quantitative assays (such as qPCR and Western blot) [77]. Because endogenous loading is naturally biosynthesized, it maintains membrane integrity and surface protein profiles, minimizing in vitro damage. Furthermore, once a production cell line is established, consistent packaging of the specific molecule is possible, enabling scalable production [78]. Endogenous loading modifies and engineers the exosome donor cells without treating the secreted exosomes, thereby better preserving their integrity and biological functions. Exosomes loaded endogenously can express specific antigenicity, thereby achieving targeted delivery [79]. Exogenous loading is a technique for directly introducing drugs into isolated exosomes, primarily utilized for the encapsulation of small nucleic acids and therapeutic agents. Common methods encompass electroporation, co-incubation, and sonication [80]. These methods can efficiently encapsulate exogenous drugs within exosomes, enhancing their in vivo stability and delivery efficiency. Compared with endogenous loading, exogenous loading is simpler to operate and has a higher loading efficiency. However, during the drug loading process, the integrity of exosomes may be compromised, necessitating additional purification steps to remove the unloaded drugs [72].

Exogenous loading often affects exosome structure and function. For example, electroporation can cause unintended vesicle aggregation and pore-induced fusion, altering vesicle size distribution or morphology, particularly at high siRNA-to-EV ratios [81]. Detergent permeabilization can introduce micropores. While effective for molecular uptake, overuse can disrupt membrane proteins or lipid raft structures [82].

In general, endogenous loading has a relatively low loading efficiency, while exogenous loading via electroporation or saponin-assisted loading can achieve higher loading efficiencies. Endogenous loading has the highest stability due to less manipulation, while exogenously loaded exosomes, especially those treated with surfactants, may increase the risk of aggregation, degradation, or functional instability during storage and transportation [83].

### The biodegradability of exosomes and its therapy on cancer

Intravenously administered exosomes have a very short plasma half-life (approximately 2 min) in mice and humans, indicating rapid systemic clearance. Within minutes, exosomes accumulate in the liver, spleen, lungs, and, to a lesser extent, the kidneys, reflecting capture by the reticuloendothelial system (RES). Exosomes are primarily internalized by macrophages (e.g., Kupffer cells in the liver and splenic macrophages) and liver sinusoidal endothelial cells via scavenger receptors (e.g., stabilin-2). Once within recipient cells (typically macrophages or endothelial cells), exosomes are transported to early and late endosomes and then to lysosomes, where their cargo (proteins, lipids, nucleic acids) is enzymatically degraded. Intratumoral (or peritumoral) delivery can bypass rapid RES clearance, thereby prolonging tumor retention and enhancing local therapeutic efficacy [84]. The small size of exosomes and their ability to fuse with receptor membranes allow them to penetrate dense ECM, including the desmoplastic stroma typical of pancreatic tumors [85]. Furthermore, engineered exosomes carrying ECM-remodeling enzymes (e.g., PH20 hyaluronidase, MMP-9, MMP-14) can degrade the matrix and improve permeability to hypovascular tumor areas and heterogeneous vesicles [86].

## Engineered exosome-based chemotherapy strategies for PC

### Loaded with gemcitabine

Gemcitabine (GEM), a pyrimidine nucleoside analogue, inhibits DNA synthesis after activation in tumor cells, leading to tumor cell death [87]. It is effective in treating a range of tumors, including lung, bladder, breast, and pancreatic cancer [88]. Nevertheless, prolonged use of GEM in cancer patients frequently leads to negative effects like bone marrow suppression and digestive system issues [89]. More critically, longterm GEM use tends to induce drug tolerance [90]. Engineered exosomes loaded with GEM have been used in tumor treatment, which can effectively alleviate drug tolerance, reduce adverse drug reactions, and enhance the therapeutic effect [91]. Exosomes from M1-macrophages, carrying GEM and deferoxamine, provide a promising approach for treating drug-resistant PC. Using electroporation drug loading technology, the encapsulation rates of GEM and deferoxamine are 6.5% and 5.7% respectively. GEM is a first line chemotherapy drug for PC and shows relatively good efficacy in clinical chemotherapy. Deferoxamine, an iron chelator, inhibits the expression of the protein RRM2 associated with drug tolerance by depleting iron, thereby enhancing the sensitivity of drug resistant PC cells to GEM. Cell experiments have demonstrated that engineered exosomes carrying drugs have better anti-cancer effects than free drugs. The survival rate of drug resistant PC cells treated with GEM + deferoxamine is 55%, while that of cells treated with drug-loaded exosomes is 29% [92].

Notably, compared with other solid tumors, the abnormal proliferation of the ECM is a prominent feature of PC, and this abnormal proliferation mediates chemotherapy resistance in PC [93]. To overcome this chemotherapy resistance, some studies [94] have incorporated GEM monophosphate and paclitaxel into exosomes sourced from mesenchymal stem cells to treat PC. The encapsulation efficiency of GEM monophosphate is 5.92%, and that of paclitaxel is 2.62%. This drug delivery system exhibits good penetrability, anti-matrix, and anti-chemotherapy resistance properties, presenting a potential method for addressing drug-resistant PC. Compared with free drugs, drug loaded exosomes show higher anti-cancer activity. Flow cytometry was used to detect the anti-cancer effects of the two drug groups. The apoptosis rate of the drug loaded (GEM monophosphate and paclitaxel) exosome group was 39.81%, while that of the free GEM plus paclitaxel group was 33.02%. In vivo experiments demonstrated that PC mice treated with drug loaded exosomes survived for 88 days, whereas those treated with free drugs survived only for 73 days. H&E staining of the major organs of the mice was performed to determine the safety profile of drug-loaded exosomes. The HE staining results showed no obvious changes in the major organs, indicating that the drug loaded exosomes are safe and reliable. To increase the concentration of drug-loaded exosomes at the tumor location, a study [95] employed exosomes derived from PC cells to carry GEM for the treatment of PC mice. The drug loading rate of GEM was 11.68%, using ultrasonic drug loading technology. More importantly, treatment with exosomes led to the disappearance of 50% of the tumors in the mice, with no obvious adverse reactions. In comparison, the tumors in mice that received free GEM swiftly reappeared once the drug was discontinued. This strategy provides valuable reference for autologous tumor treatment using autologous exosomes as drug carriers.

### Loaded with Paclitaxel

Paclitaxel (PTX) is a natural anti-cancer drug extracted from plants [96]. PTX exerts its anti-tumor effect by blocking the mitosis of tumor cells [97]. It is primarily utilized for treating breast cancer, ovarian cancer, advanced non-small cell lung cancer, and PC [98]. However, PTX has certain drawbacks. It can cause hematological toxicity, neurotoxicity, and affect cardiac activity [99]. Extended PTX usage might result in symptoms like neutropenia, peripheral neuropathy, and arrhythmia [100]. Moreover, PTX has low solubility in water [101]. Therefore, in clinical treatment, polyoxyethylene castor oil and ethanol need to be added. Unfortunately, polyoxyethylene castor oil may cause allergic reactions [102]. Engineered exosomes loaded with PTX for tumor treatment can effectively reduce the adverse reactions of PTX and enhance its therapeutic efficacy.

Mesenchymal stem cells (MSCs) hold the capacity to target the TME. Research [103] has revealed that the SR4987 mouse bone marrow mesenchymal stem cell line shows a strong resistance to PTX’s cytotoxic effects. Approximately 85% of MSCs can survive and secrete PTX-rich exosomes under high dose PTX exposure, indicating that MSCs can serve as a “factory” for producing PTX-rich exosomes. To develop drugs with higher tumor targeting properties and reduce the adverse reactions of PTX, in this study, MSCs were treated with high-dose PTX to secrete PTX-rich exosomes. Infrared spectroscopy analysis demonstrated that PTX binds to exosomes. The results indicated that the PTX-loaded exosomes exert a dose dependent inhibitory effect on the proliferation of PC cells. When the protein concentration of PTX-loaded exosomes ranges from 0.047 to 0.095 mg/mL, the growth inhibition rate of PC cells reaches 50%. When the exosome protein concentration is 0.38 mg/mL, the growth inhibition rate of PC cells is 80%. However, the encapsulation efficiency was not discussed. The research team further discovered that MSCs isolated from Human gingiva also have a high tolerance to PTX. Under high dose PTX exposure, gingival MSCs can survive and release PTX-rich exosomes, with the loading amount of PTX in exosomes being approximately 36.8 ng/mL. In this experiment, the anti-pancreatic cancer activity of MSCs was detected through a co-culture system. MSCs treated with PTX were co-cultured with PC cells as the experimental group, while MSCs not treated with PTX were co-cultured with PC cells as the control group. After 24 h of co-culture, trypan blue staining indicated no toxicity in cancer cells from the control group, while numerous blue-stained dead cancer cells were seen in the experimental group [104].

Improving the tumor-targeting capability of exosomes by altering the targeting molecules on their surface is an effective method for the accurate delivery of chemotherapy drugs. The functional ligand RGD, a polypeptide composed of repeated sequences of arginine, glycine, and aspartic acid, exhibits a high affinity for integrin αvβ3, which is highly expressed on PC cells. By attaching RGD to the surface of exosomes from PC cells, their targeting potential can be notably increased. Afterward, PTX was incorporated into RGD-modified exosomes to treat PC in a mouse model. In this study [105], ultrasound-mediated drug delivery technology was employed, achieving a drug delivery efficiency of 36.8% for PTX. The results demonstrated that, compared to free PTX, the RGD-modified drug-loaded exosomes could effectively penetrate the TME of PC and target tumor cells. Consequently, they effectively inhibited tumor growth in the PC mouse model with fewer chemotherapy-related adverse reactions.

### Loaded with doxorubicin

Doxorubicin (DOX) is an antibiotic used to fight tumors. It primarily works by entering cells and attaching to DNA, which prevents the synthesis of nucleic acids [106]. DOX serves as a treatment option for several tumors, including leukemia, sarcoma, breast cancer, and ovarian cancer [107]. However, it has adverse reactions including bone marrow suppression and cardiotoxicity [108]. Recently, studies [109] have used exosomes loaded with DOX for antitumor treatment, achieving good efficacy and reducing the adverse reactions of chemotherapy. One study [110] analyzed the efficiency of exosome secretion from different cell types, the loading efficiency of DOX into exosomes from various sources, and the anti-pancreatic cancer effects of exosomes loaded with different drugs. In this study, PC cells, pancreatic stellate cells, and macrophages were cultured in a DOX medium to secrete exosomes rich in DOX. Then, the drug loaded exosomes in the cell supernatant were collected by ultra-centrifugation. The protein concentration of exosomes revealed that PC cells secreted the highest amount, with macrophages and pancreatic stellate cells following. PC cell-derived exosomes showed the greatest loading efficiency at 14.06 ng/µg, compared to macrophages at 7.27 ng/µg and pancreatic stellate cells at 3.99 ng/µg. The anti- pancreatic cancer activity of exosomes from different sources was detected by flow cytometry. Exosomes derived from macrophages and loaded with DOX showed the highest anticancer activity (apoptosis index of 80.1%), followed by those from pancreatic stellate cells (apoptosis index of 73.2%) and PC cells (apoptosis index of 65.3%). This study indicates that exosomes have donor-cell specific differences, which may affect their therapeutic effects as drug carriers. It provides an important theoretical basis for the construction of a safe and efficient drug delivery system.

**Fig. 3:**
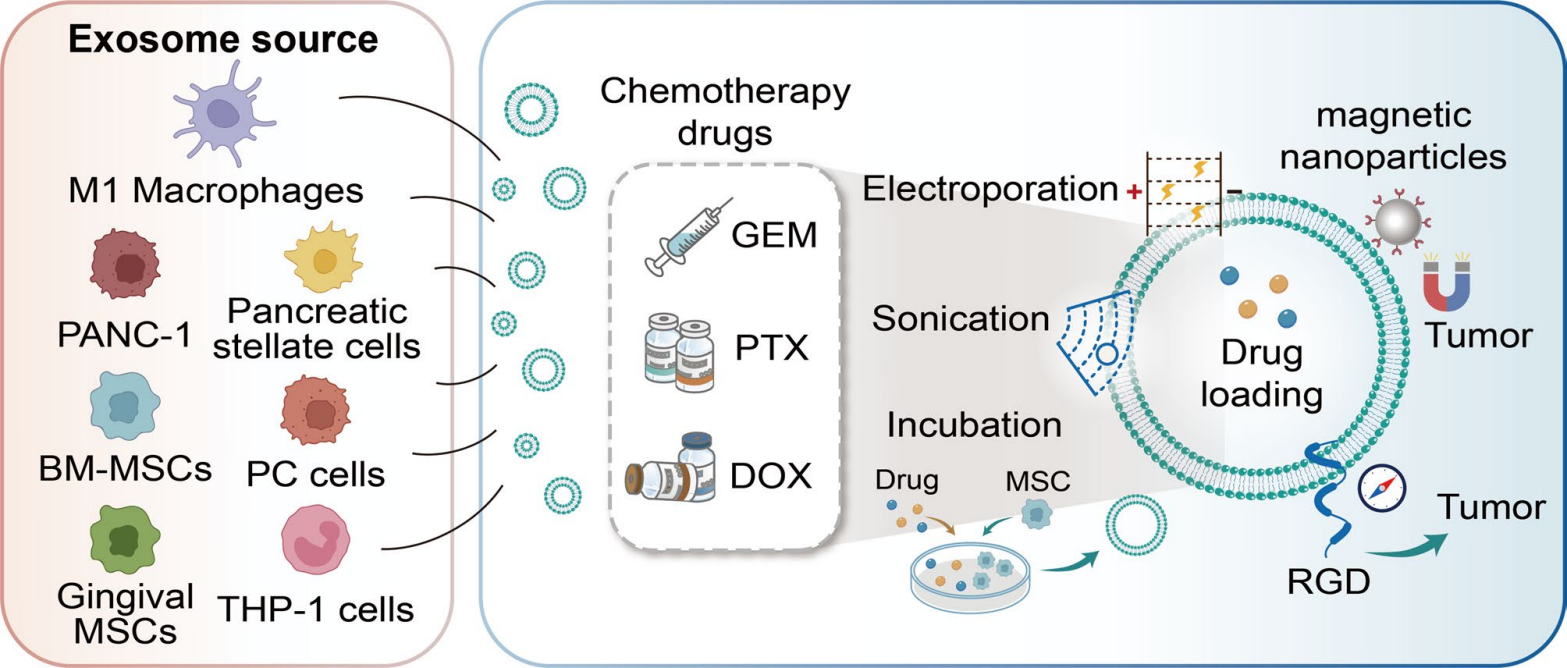
Applications of engineered exosome-based chemotherapy strategies. The central focus is the delivery of chemotherapeutic agents including Gemcitabine(GEM), Paclitaxel (PTX), and Doxorubicin (DOX) through exosomal carriers

The combination of exosome drug delivery systems with various therapies provides important references for the exosome-based drug treatment of PC. A study [111] investigated a drug delivery system that integrates chemotherapy, gene therapy, and photothermal therapy. HE staining of tissue sections and blood analysis were performed on the experimental mice to evaluate its in vivo biosafety. According to the results, this drug delivery system is both safe and feasible, exhibiting low tissue toxicity. This drug delivery system consists of exosomes loaded with DOX and magnetic nanoparticles modified with molecular beacons. An external magnetic field and near-infrared radiation induce the drug delivery system to function. Molecular beacon-modified magnetic nanoparticles can direct drug-loaded exosomes to gather at the tumor location using an external magnetic field. Once the exosomes gather at the tumor location, near-infrared radiation is used to create local hyperthermia and initiate the release of the contents within the exosomes. Released molecular beacons are able to target miRNA-21 for both imaging and gene silencing, while DOX serves to eliminate cancer cells. Recent studies have also loaded chemotherapeutic drugs such as elastin and oxaliplatin into exosomes for tumor chemotherapy. Loading chemotherapeutic drugs into exosomes (Fig. 3) is beneficial for improving drug targeting, increasing efficacy, and reducing adverse reactions. As shown in Table 1, we summarize the successful applications of engineered exosome-based chemotherapy strategies in PC.

## Engineered exosome-based immunotherapy strategies for PC

### Immunotherapy with immune checkpoint inhibitors

Immune checkpoint inhibitors (ICIs) are capable of blocking inhibitory signaling pathways, which boosts T cell activation and strengthens the body’s immune response against tumors [112]. ICIs primarily target negative immune checkpoint molecules, including cytotoxic T lymphocyte-associated antigen 4 (CTLA-4), programmed death 1 (PD-1), and programmed death-ligand 1 (PD-L1), which are involved in strengthening the immune response and postponing tumor advancement [113]. Numerous experimental studies have shown that exosomal PD-L1 is significantly elevated in the serum of PC patients, suggesting a poor prognosis and clinical importance [114, 115]. Exosomal PD-L1 diffuses to the periphery through the circulation and directly binds to PD-1 on the surface of T cells, inhibiting T cell activation (TCA) and inducing their depletion. Exosomal PD-L1 systematically inhibits T cell activity by mimicking the function of PD-L1 on the cell surface, weakening the therapeutic efficacy of anti-PD-1/PD-L1 antibodies. In chronic antigen-exposed environments (e.g., TME) where T cells highly express PD-1, exosomal PD-L1 exacerbates their loss of function, leading to failure of the antitumor immune response [116, 117].

A recent preclinical study [118] cultivated and collected CD133-targeted exosomes, which were then loaded with PD-L1 siRNA to treat a murine model of metastatic PC. Following intravenous administration, the metastatic liver and pancreatic tissues exhibited greater overall radiation efficiency, indicating better targeting than other control materials. There was a notable increase in pro-apoptotic markers (BIM and c-caspase 3) and a slight decrease in anti-apoptotic markers (Mcl-1 and Bcl-xL), validated through multiple techniques such as real-time polymerase chain reaction, Western blot analysis, and immunohistochemistry (all *p* < 0.05). The findings confirmed that CD133-tEx with PD-L1 siRNA can exert strong anti-cancer effects by selectively binding to CD133-positive PC cells and suppressing PD-L1 expression. Another innovative study [119] discovered that the key subunit of exosomal complex Exo70 can promote PC metastasis by regulating the secretion of tumor exosomes, and can induce immune evasion in PC cells by increasing the expression of exosomal PD-L1. They also confirmed that knocking down Exo70 or using an Exo70 inhibitor blocked MVBs in cells, reducing the release of tumor exosomes and PD-L1 expression, thereby inhibiting immune evasion dependent on the tumor exosome pathway. This implies that Exo70 treatment could potentially increase the efficacy of PD-L1 immunotherapy and present a new strategy for PC therapy.

Although initially used as monotherapy, an increasing number of patients are now receiving combined immune checkpoint blockade, primarily due to the non-redundant mechanisms of action of single-agent therapies typically leading to higher response rates. Additionally, immune checkpoint therapy has been combined with chemotherapy, radiotherapy, and other immunotherapeutic agents to maximize clinical efficacy [120]. One study [121] isolated exosomes from bone marrow MSCs, loaded them with siRNA targeting KRASG12D, and analyzed their effects on tumor cell KRAS signaling, FAS epigenetic regulation, and the immune microenvironment, validating their synergistic effect with anti-CTLA-4 therapy. They employed a 3 + 3 dose-escalation and accelerated titration design, enrolling advanced PC patients in a human phase I clinical trial to assess safety, tolerability, and biomarker changes, providing a safe and targeted treatment option for KRAS-mutant-driven PC. By influencing the tumor immune microenvironment, this research creates new possibilities for combined immune checkpoint therapy, which could lead to better patient prognoses. Similarly, another research team [122] created a dual-delivery biological system based on bone marrow MSCs exosomes, called iEXO-OXA, which was loaded with galectin-9 siRNA using electroporation and had its surface modified with an oxaliplatin prodrug. This system enhances tumor targeting, facilitates drug concentration at the tumor location and boosts immune cell death responses by altering macrophage polarization, attracting cytotoxic T cells, and reducing regulatory T cells, demonstrating significant efficacy in PC treatment and providing a new strategy for PC immunotherapy.

### Immunotherapy with cancer vaccine

Vaccine therapy represents another form of immunotherapy. It functions by revealing cancer-associated antigens to the immune system, thus stimulating T cells and strengthening the anti-tumor immune response [123]. Therapeutic cancer vaccines can help maintain a sufficient level and function of immune effector cells. The goal of this active immunotherapy is to provoke an anti-tumor immune response targeting tumor associated antigens (TAAs) or tumor- specific antigens (TSAs) [124]. Over the past four decades, cancer vaccines targeting various malignancies have been the subject of pre-clinical and clinical research. To date, a variety of traditional vaccines for PC are under development, including those based on cells, microorganisms, exosomes, proteins, peptides, and DNA [125]. Among them, exosome-based PC vaccines are an emerging area of research. This is mainly because, although all cells produce exosomes, tumor cells are particularly active in exosome production. The production, release, and use of exosomes by tumor cells promote tumor growth, resulting in higher exosome concentrations in the plasma or other body fluids of cancer patients than in those of healthy individuals [126]. Presently, significant research efforts are directed at uncovering the mechanisms through which tumor-derived exosomes aid in tumorigenesis.

Tumor-derived exosomes, known as “TEXs”, are nanoscale vesicles enclosed by a lipid bilayer. They serve as a communication network for autocrine, paracrine, and contact-dependent signal transduction among various cells within TME [127]. These exosomes have the ability to reprogram diverse cells in the TME, including immune cells. The ability of TEXs to suppress anti-tumor immunity and promote immune evasion mainly consists of (1) carrying immunosuppressive PD-L1 on their surface and suppressing activated T cells (2) reprogramming tumor-infiltrating immune cells to an immunosuppressive phenotype (3) inhibiting NK cell function (4) promoting metastatic niches by recruiting and educating immune cells [128]. Given their unique biological origin, potential as non-invasive cancer biomarkers, and capacity to regulate immune cell functions and suppress anti-tumor responses, TEXs have attracted extensive attention [129]. Up to now, TEXs have been shown to be effective anti-cancer vaccines in animal models, capable of inducing antigen-specific T-cell and B-cell responses and functioning as powerful vaccine-promoting carriers [130]. However, due to the immunosuppressive effects induced by TEXs and their limited immunogenicity, using TEXs alone often leads to poor anti-tumor immune effects in vivo. To improve the effectiveness of TEX vaccination, tumor cells and TEXs can be modified (either genetically or non-genetically) to increase the presence of tumor antigens, microRNAs, and immunostimulatory molecules in TEXs [131]. This enrichment aims to directly enhance tumor cell clearance or exert cytotoxic effects in synergy with immune cells. Zhou et al. developed an engineered exosome vaccine (spMEXO) derived from immunogenic dying tumor cells and enhanced anti-tumor immunity by modifying MART-1 peptide and loading CCL22 siRNA. The vaccine was targeted for delivery through the lymphatic system after intramuscular injection and effectively overcame the stromal barrier of PDAC; whereas, it significantly inhibited tumor progression in both prophylactic and combination chemotherapy treatment models, providing a multifunctional platform for PC immunotherapy [132]. Therefore, engineered TEXs as anti-tumor vaccines not only inherit tumor antigens and molecules but also exhibit stronger anti-tumor immune activity and reduced pro-tumor effects [133].

### Immunotherapy with adoptive cell therapy

Adoptive cell therapy (ACT) is an immunotherapy approach that employs the patient’s own immune cells, including T cells, to tackle diseases. Typically, these immune cells are harvested, expanded, and genetically altered to boost their effectiveness in attacking cancer cells. T cells are engineered to target tumor cells by recognizing their unique molecular characteristics. ACT encompasses various treatment modalities, including tumor-infiltrating lymphocyte (TIL) therapy, genetically engineered T cell therapy (e.g. T cell receptor (TCR)-engineered T cells, chimeric antigen receptor (CAR)-T cell therapy), CAR - natural killer (NK) cell therapy, and cytokine- induced killer (CIK) cell therapy [8]. However, a major challenge in treating PC, particularly with CAR-T cell therapy, is the absence of appropriate tumor-specific antigens. Antigen selection is a crucial barrier to the implementation of CAR-T and TCR-T strategies in PC. This is due to the fact that many studies focus on tumor-associated antigens that are unevenly distributed on tumor cells, potentially raising the risk of on-target, off-tumor toxicity [134].

Through CAR-T cell therapy, a patient’s T cells are genetically engineered to specifically seek out and eliminate cancer cells. The success of CAR-T cell therapy in blood-related cancers has generated enthusiasm for its potential use in treating solid tumors such as PC [135]. However, CAR-T cell therapy confronts significant limitations, such as severe toxicity, antigen escape, and limited tumor infiltration, all of which pose major challenges to clinical translation and large-scale production [136]. To address these limitations, innovative strategies are being developed to utilize exosomes derived from CAR-T cells to inhibit tumor growth. This approach presents a potentially safer and more effective option for immunotherapy, which has garnered extensive attention in the area of oncology research. These exosomes are capable of being engineered to carry diverse cargos like tumor-targeting receptors, siRNAs, or cytokines, thereby augmenting their anti-cancer effects [137]. Recently, the multi-target CAR strategy has been demonstrated to be feasible for overcoming the burden of tumor recurrence [138]. Exosomes derived from multi-target CAR-T cells may possess multiple targeting sites on their surface, which is ascribed to the heritability of exosomes. Thus, exosomes engineered from CAR-T cells are likely to become a more efficient and less risky treatment choice for patients [139]. Moreover, adjusting the variable binding region and hinge region of the CAR on the exosome surface, as well as adjusting the affinity, can further strengthen their binding ability to tumor cells. Nevertheless, the efficacy of these exosomes produced by engineered cells must be verified in future research and preclinical models. Apart from their heritability, the cargo-loading capacity of exosomes is also of crucial importance. Therefore, engineering exosomes to address current challenges is a more suitable technological approach [140]. Similarly, chemotherapeutic drugs that induce high expression of antigens on the surface of tumor cells (such as ATRA) can be incorporated into exosomes released by CAR-T cells [141]. Another strategy to enhance the anti-tumor effect is to load exosomes with microRNA or RNA interference (RNAi) molecules to achieve a combined gene and immunotherapy effect on tumors [136]. In the latest studies, researchers depleted microRNA in exosomes through lysis and ultracentrifugation, thereby enhancing the immunogenicity of exosomes extracted from the supernatant of cultured PANC-1 cells. Remarkably, proteins within microRNA-depleted exosomes increased the tumor-killing effectiveness of DC/CIK cells against PC cells, implying that exosomes engineered from PC cells could be a viable immunotherapy method for PC [142].

### Combination immunotherapy

The immunotherapy for PC has shown a glimmer of hope, but the significant clinical benefits remain elusive. With each failure of a late-stage trial, it becomes increasingly apparent that monotherapy is unlikely to succeed in PC, and the most promising approach in the short term is the combination of immunotherapy with other treatments [143].

Several years ago, Professor Li’s team [144] explored the potential of combining DC vaccines loaded with tumor-derived exosomes with GEM and/or ATRA and/or Sunitinib to enhance the therapeutic efficacy of PC. Using the UNKC6141 PaCa cell line, the experiments involved weekly intravenous injections of UNKC6141 TEX-loaded DCs, along with GEM and/or ATRA and/or Sunitinib. The findings showed that vaccinating mice with DC-TEX notably increased their survival, and incorporating GEM along with other chemotherapy drugs led to more activated T cells in the tumor, further lengthening survival.Given that ATRA, GEM, and Sun act at different stages of maturation and activation of MDSCs, it is anticipated that these chemotherapy drug combinations could synergize with the therapeutic effects of DC-TEX vaccination. The integration of DC-TEX vaccination with chemotherapy aimed at MDSCs is among the most promising strategies for PC treatment, potentially boosting tumor cure rates significantly, even post-metastasis.

**Fig. 4:**
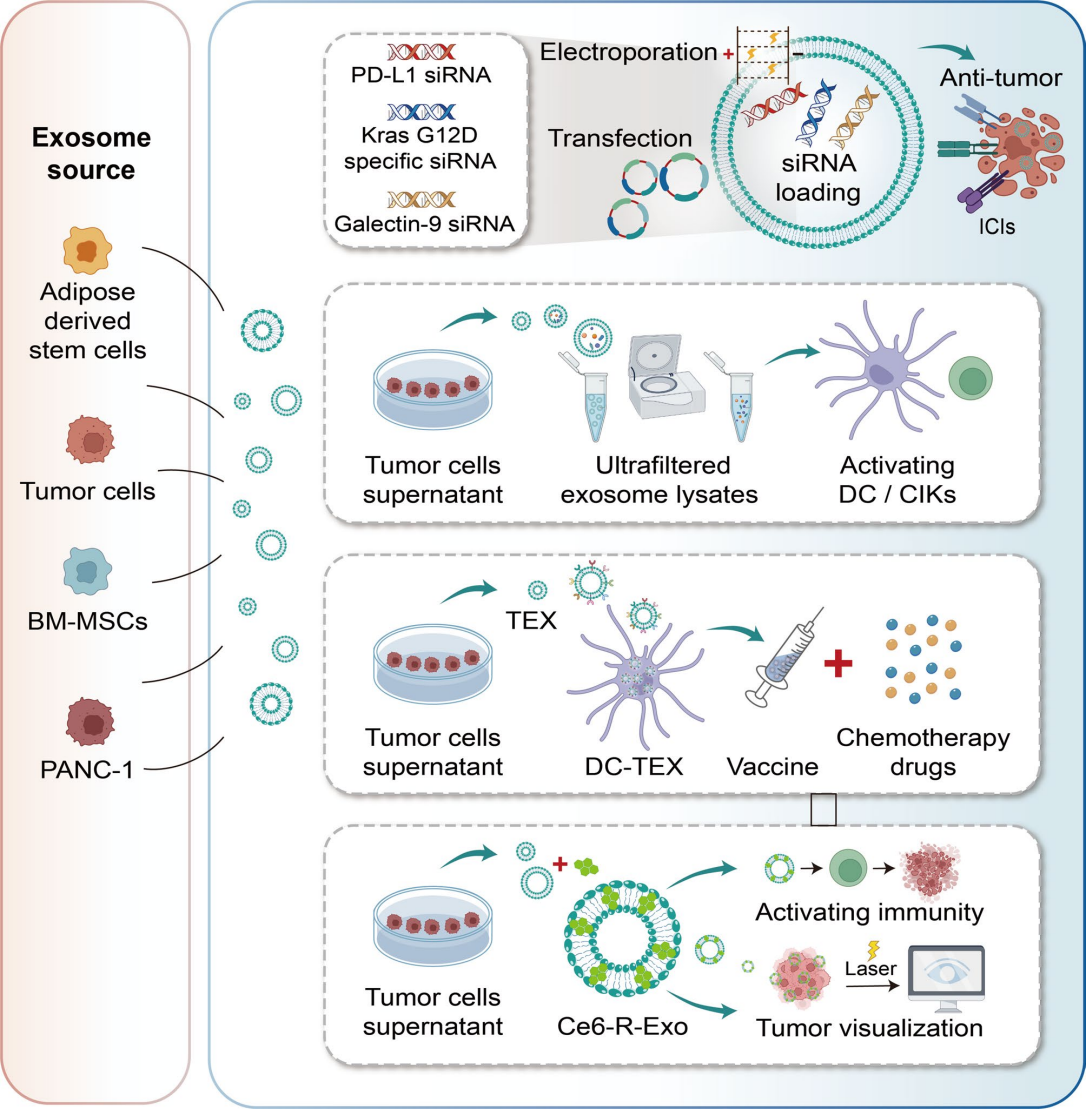
Applications of engineered exosome-based immunotherapy strategies, including exosome-based immune checkpoint inhibitors (ICIs), tumor vaccines, adoptive cell therapy (ACT) and potential combination immunotherapy. In this figure, we summarize the sources of exosomes, valid cargos and engineering methods of some representative immunotherapy experiments

A more recent representative example is the work of Professor Jang’s team [145], who utilized chlorin e6 photosensitizer-modified tumor-derived recombinant exosomes (R-Exo). Using photoacoustic imaging, Chlorin e6-modified R-Exo (Ce6-R-Exo) can be observed and it efficiently creates reactive oxygen species in tumor cells when irradiated with a laser. Furthermore, Ce6-R-Exo augments the capability of immune cells to discharge cytokines, showing that these engineered exosomes can also be utilized as agents for immunotherapy. This innovative approach combines photoacoustic imaging-guided photodynamic therapy with immunotherapy using tumor-derived Ce6-R-Exo to treat PC. As shown in Fig. 4; Table 2, we summarize the successful applications of engineered exosome-based immunotherapy strategies in PC.

## Engineered exosome-based gene therapy strategies for PC

### Gene transfer

The therapeutic modality of gene transfer therapy for PC principally involves the utilisation of diverse strategies and tools for the introduction of tumour suppressor genes (such as p16, p21, p53, Smad4/DPC4, etc.), anti-angiogenic genes (such as endostatin), apoptosis -related genes (such as TRAIL, TNF), and suicide genes [146]. P21-activated kinase 4 (PAK4) is oncogenic when overexpressed and is associated with increased proliferation, survival, migration, and metastasis of PC cells. A study [147] showed that siRNA was encapsulated into exosomes derived from PC cells via electroporation, and co-localization was confirmed in PANC-1 cells using fluorescence microscopy. The results indicated that these exosomes could reduce PAK4 expression and decrease tumor growth both in vivo (intratumoral injection) and in vitro. The efficacy of PAK4 as a therapeutic target for PC was demonstrated by the finding that its inhibition significantly prolonged the survival of mice bearing PC. A few years ago, David Novo et al. found that exosomes produced by tumor cells expressing mutant p53 (mutp53) mediate the invasive/migratory acquisition of mutp53 in intercellular transfer by increasing Rab-coupling protein (RCP)-dependent integrin cycling in other tumor cells, and also affect integrin in normal fibroblasts transport to promote the deposition of pro-invasive ECM rich in the pro-invasive microenvironment [148]. And the latest experiments confirmed that exosomes secreted from OBP-702 (p53-armed lysogenic adenovirus) treated PC cells (called Exo702) could efficiently activate DCs and induce systemic anti-tumor immunity. Mainly including (1) Exo702 significantly upregulated DC maturation markers (CD86/CD80/CD83) and IFN-γ secretion, (2) OBP-702 intratumorally increased the proportion of mature DCs (CD86+/CD11c+/MHC-II+) in the draining lymph nodes by 2-fold, accompanied by CD8 + T-cell infiltration. (3) Exo702 monotherapy induced distal tumor regression (“distal effect”) immediately, with a 40% inhibition rate. (4) Exo-702 maintained Durable anti-tumor immune memory 28 days after treatment. This study reveals for the first time that p53-lysovirus can be transformed into a DC-activated vector by modifying the components of tumor exosomes, providing a new strategy for engineered exosomes combined with p53 gene therapy [149]. Smad4 or DPC4 belongs to a group of signal transduction proteins phosphorylated and activated by transmembrane serine-threonine receptor kinases in response to transforming growth factor beta (TGF-β) signaling through multiple pathways. This gene acts as a tumor suppressor gene and inactivation of smad4/DPC4 is most prominent in PC [150]. The 2011 phase II trial of the combination of cetuximab, GEM, and oxaliplatin followed by adjuvant chemoradiotherapy with cetuximab for locally advanced (T4) pancreatic adenocarcinomas demonstrated a correlation between immunostaining for Smad4(Dpc4) and the pattern of disease progression, suggesting that the expression of Smad4(Dpc4) has a predictive biomarker of value and may lead to personalized treatment strategies for patients with localized PC [151].

As with other solid tumours, the growth and metastasis of PC are contingent on angiogenesis. PC exhibiting high expression levels of vascular endothelial growth factor (VEGF), a potent pro-angiogenic cytokine, have been associated with increased metastasis incidence and poorer prognosis. Consequently, multiple experimental anti-angiogenic strategies are under investigation to mitigate tumor progression, dissemination, and vascularization in PC [147, 152]. However, to date, there has been no successful report on gene therapy for PC by introducing anti-angiogenic genes through engineered exosomes. Only a few similar experiments are underway. For example, the research of Professor Wang’s team [153] demonstrated that the microRNA, miR-29b, when present within exosome preparations derived from PC cells, exhibited the capacity to confer protection to human umbilical vein endothelial cells (HUVECs) from the process of PC-induced angiogenesis. This protective effect was attributed to the attenuation of the expression levels of two specific genes, ROBO1 and SRGAP2, within the HUVECs. Professor Chen’s study [154] indicated that exosomal extracts from hypoxic PC cells exhibit a significant enrichment of microRNA-30b-5p, which has been shown to promote angiogenesis by inhibiting GJA1. This finding suggests that microRNA-30b-5p could serve as a potential diagnostic biomarker for PC.

Notably, mesenchymal stem cell-derived exosome (MSC-exosome) therapy has emerged as a promising tool for tumour-targeted therapy due to the following properties: high stability, low immunogenicity, excellent biocompatibility, prolonged circulation time and homing properties [155]. It can be hypothesised that the combination of MSC-exosomes with other therapeutic modalities could result in a significant enhancement in the efficacy of cancer treatment. It has been demonstrated by preceding experiments that the engineering of MSC-exosomes has the capacity to enhance their advantages and tumour-suppressive effects, thus overcoming the limitations associated with pro-tumour actions. As we have previously outlined in some studies related to chemotherapy and immunotherapy, utilizing these engineered exosomes as nanocarriers to deliver non-coding RNA or their inhibitors, as well as anti-cancer drugs, represents an emerging and promising strategy for cancer treatment [156].

Tumor necrosis factor-related apoptosis-inducing ligand (TRAIL) is a promising anti-cancer therapeutic agent, attracting significant attention due to its specificity towards cancer cells and potent anti-tumour activity. Nevertheless, the accelerated elimination of TRAIL from the bloodstream, in conjunction with its brief plasma half-life, serves to curtail its therapeutic potency. To overcome these limitations, strategies for directly delivering the TRAIL gene to tumors are being explored. For instance, PEI25Pyr50%, a non-viral vector, can be used to transfect plasmids encoding TRAIL/GFP into MSCs, generating TRAIL-engineered MSCs. The study demonstrated that the exosome derived from TRAIL-engineered mesenchymal stromal cells (MSCs) exhibited tumour-homing ability in both in vitro and in vivo models. Furthermore, the exosome demonstrated effective anti-tumour activity in a mouse melanoma model [157]. Regrettably, there have been no reports on the efficacy of TRAIL-engineered exosomes in PC treatment to date. It is gratifying that recent research [158] indicates that the combination of TRAIL gene therapy and GEM, the first-line chemotherapy drug for PC, can induce apoptosis in human PC cell lines in vitro. Moreover, MSCs themselves can inhibit PC cells, and this effect can be enhanced by transfecting cells expressing TRAIL with death receptors [159]. In a recent study, Ella Rimmer’s team [160] found that exosomes derived from PC cells can confer resistance to GEM and TRAIL treatment. This suggests that removing these exosomes during treatment can improve the cells’ response to GEM and TRAIL, thereby enhancing treatment efficiency. Similarly, Daniela Klimova’s research [161] successfully designed periodontal ligament stem cell-derived exosomes loaded with GEM. These exosome samples were engineered to express a suicide gene fusion of yeast cytosine deaminase: uracil phosphoribosyltransferase. The product of the suicide gene converts the non-toxic prodrug 5-fluorocytosine (5-FC) into the highly toxic chemotherapeutic drug 5-fluorouracil (5-FU) inside recipient cancer cells. The present study investigates the inhibitory effect of the conversion of 5-FC to 5-FU on cancer cell growth. The results demonstrate that this conversion exerts an additional inhibitory effect, thereby suppressing the proliferation of PC cell lines in vitro.

### Gene invalidation

Gene invalidation therapy for PC mainly focuses on targeting oncogenes such as KRAS, as well as new methods of directly targeting non-coding RNAs (ncRNAs).It is evident that the GTPase mutant form of KRAS exerts a pivotal role in the development of PC [162]. Research on this challenging therapeutic target has centred on the use of exosome-derived small interfering RNAs (siRNAs) or short hairpin RNAs (shRNAs) that are specific to KRAS G12D. These siRNAs and shRNAs are delivered to the target cells via exosome-rich conditioned media derived from normal fibroblast like mesenchymal cells. The approach is contingent upon CD47-dependent phagocytosis, which is instrumental in safeguarding and augmenting the direct and specific targeting of the oncogene KRAS in tumors. This engineered exosome strategy has demonstrated the ability to inhibit cancer progression and significantly improve overall survival in multiple PC mouse models [163].

In addition to conventional gene transfer methodologies employing carrier DNA (synthetic or viral vectors), an alternative approach entails the direct targeting of tumour-suppressing non-coding RNAs (ncRNAs), thereby bypassing DNA and its transcription process. The two main types of small RNAs with therapeutic significance are microRNAs and interfering RNAs, such as siRNAs and shRNAs. These ncRNAs play roles in many aspects, especially in post-transcriptional gene silencing, it has been hypothesised that they have the potential to act as carriers for gene therapy in the treatment of various diseases, including cancer [164]. However, the controllable expression of ncRNA-based therapies, particularly in terms of level, time, and location, remains a significant challenge and the main obstacle to ncRNA based fundamental treatments. Fortunately, the advancement of engineered exosomes brings hope. The loading of tumour-suppressing ncRNAs into engineered exosomes enables the precise, temporal, spatial and dosage-controlled delivery of therapeutic ncRNAs to tumour sites [165]. Ding [166] isolated exosome samples were extracted from the superior portion of umbilical cords. Subsequently, these samples were loaded with microRNA-145-5p and then injected into mice via an intratumoral route. This microRNA has been linked to a decrease in the number of PC cells, with studies indicating that it exerts its anti-tumour effect by regulating the TGF-β/Smad3 signalling pathway. Han’s data show that microRNAs originating from the tumour-associated stroma (TAS) can be transferred to adjacent PC cells via exosome, thereby impeding the proliferation of tumour cells [167]. This highlights the potential anti-tumor ability of modifying exosomes secreted by TAS cells with tumor suppressive genetic materials. These engineered exosomes have therapeutic implications for unresectable PC. In a similar manner, the encapsulation of miR-34a by exosome has been demonstrated to effectively traverse cell membranes, thereby inducing a decrease in the expression of the target gene Bcl-2. A series of in vitro and in vivo experiments have been conducted in order to ascertain the effects of exosome-mediated miR-34a treatment on the growth of PC cells. The results of these experiments have confirmed that this treatment significantly inhibits the growth of PC cells and induces cancer cell apoptosis by regulating the expression of apoptosis-related genes [168], providing a promising strategy for PC treatment.

The latest and most innovative gene editing technology, CRISPR/Cas9, has proven effective in targeting specific parts of the genome, yielding encouraging results [169]. Although CRISPR/Cas9 represents a new technological revolution, the specific and safe in-vivo delivery of this editing tool remains challenging. Moreover, it is currently impossible to ensure that CRISPR/Cas9 can target al.l cells in the target population. However, there have been reports on CRISPR/Cas9 loaded exosomes, which can address these issues and specifically activate necrosis within tumors [170]. In the report by Kathleen M.McAndrews [171], a non-viral CRISPR/Cas9 delivery system was described, the basis of which was engineered exosome. It has been demonstrated that non-autologousosomes have the capacity to encapsulate CRISPR/Cas9 plasmid DNA using common transfection reagents to target the mutant KRAS G12D oncogene in PC.This approach has been established to reduce cancer cell proliferation and inhibit the growth of PC cells in both subcutaneous and orthotopic models.

**Fig. 5:**
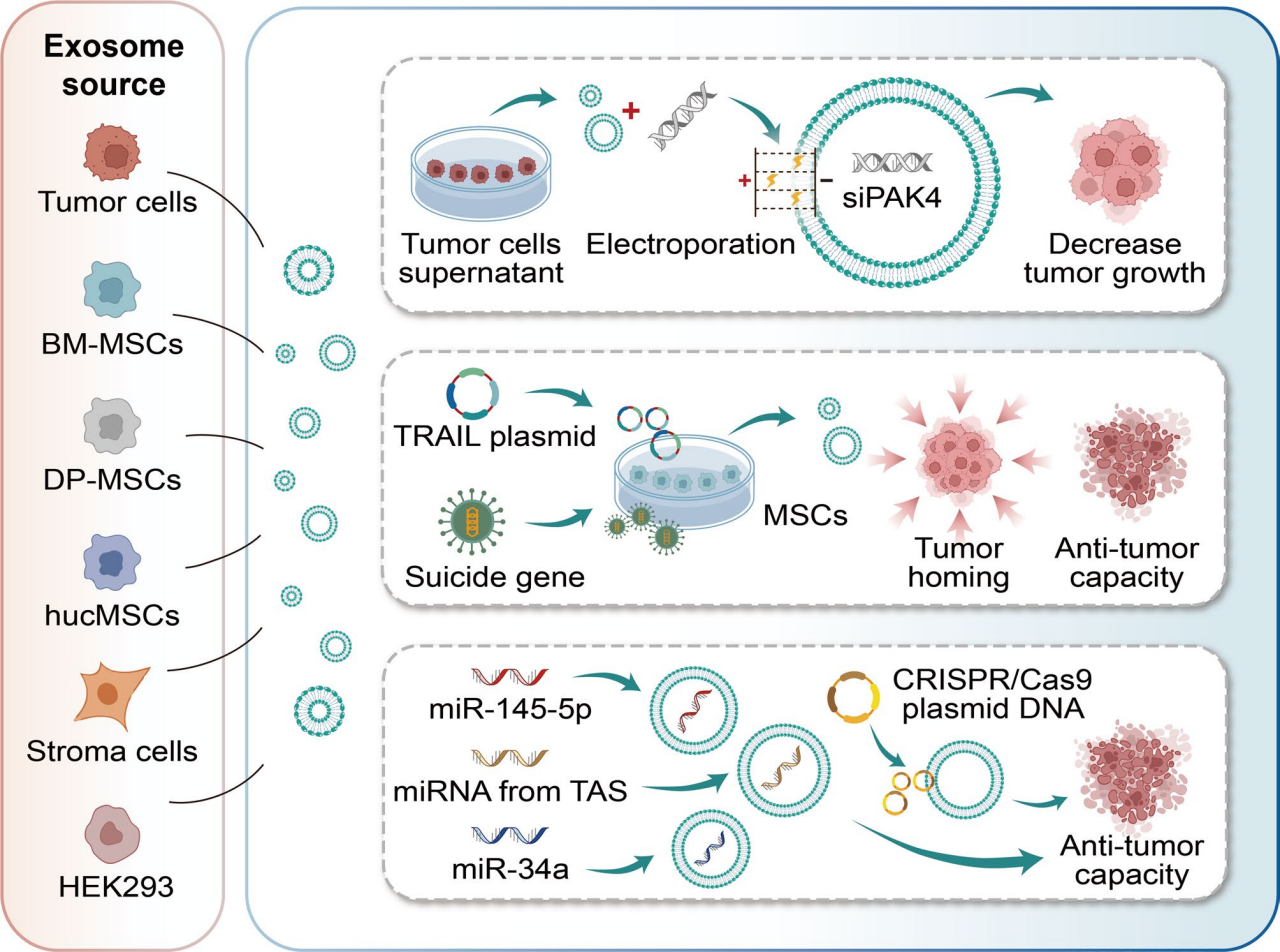
Applications of engineered exosome-based gene strategies. It can be broadly categorized into the implementation of gene transfer systems (introducing tumor suppressor genes, anti-angiogenic genes, apoptosis -related genes, and suicide genes) and gene invalidation strategies (targeting oncogenes and non-coding RNAs). In this figure, we summarize the sources of exosomes, valid cargos, engineering methods and therapeutic effects of some representative gene therapy experiments

To summarize, notwithstanding a number of challenges, the therapeutic efficacy of ncRNA and CRISPR/Cas9 have been substantiated. The development of engineered exosome technology has the potential to bridge the gap between pre-clinical and clinical trials for ncRNA and CRISPR/Cas9 gene therapy. This is due to their high tumour-targeting ability and biocompatibility [15]. Despite the absence of gene therapy drugs for PC on the market at present, a significant number of clinical trials are currently underway, particularly those in the active recruitment phase or that have recently been completed [172]. An increasing number of experts believe that gene therapy is feasible for PC and may have synergistic anti-tumor activity with standard treatment and immunotherapy, just as what we show in Fig. 5. The growing pre-clinical and clinical data pave the way for improving the treatment of PC patients, showing broad development potential [173].

## Engineered exosome-based targeted therapy strategies for PC

### Targeting exosome-secreted proteins

Effective biomarkers that can guide treatment and have limited adverse effects have become an attractive research topic in the field of cancer diagnosis and treatment [174]. Exosomes that come from cancer cells are extracellular vesicles that display the molecular characteristics of those cells. They can act as a reliable source for potential biomarkers, such as proteins and nucleic acids, in non-invasive cancer diagnosis and prognosis [175]. Tumor-derived exosomes preferentially home to their cells of origin (even within the metastatic microenvironment), delivering diverse cargo to heterogeneous subpopulations within the tumor [176]. This intrinsic targeting facilitates the infiltration of multiple tumor cell subtypes, even in areas difficult to reach by synthetic nanocarriers. Beyond cancer cells, exosomes can also be engineered to deliver immunomodulatory agents (e.g., galectin-9 siRNA/exosomal oxaliplatin), thereby reprogramming TAMs from an M2 to an M1 phenotype and reshaping the immune landscape of heterogeneous cell populations [177]. Exosomes containing proteins may participate in tumor development and progression via several signaling pathways, including epithelial-mesenchymal transition (EMT), invasion, and metastasis [178]. From a clinical standpoint, exosomal proteins are vital in conveying oncogenic potential or treatment resistance to recipient cells, which could aid in shaping treatment strategies [179].

Fan [180] determined through comparative proteomics that compared with the exosomes of PC cell lines MIA PaCa-2 and BxPC-3, which have lower chemotherapy resistance, Ephrin type-A receptor 2 (EphA2) is overexpressed in the exosomes of PANC-1. Furthermore, reducing EphA2 levels in PANC-1 cells hampers their capacity to convey exosome-mediated chemotherapy resistance to MIA PaCa-2 and BxPC-3. This finding indicates that exosomal EphA2 expression can convey chemotherapy resistance and might act as a minimally invasive predictive biomarker for assessing treatment response in PC. Targeted inhibition of exosomal EphA2 holds significant therapeutic implications.

Hirokazu Kimura’s research [181] has found that cytoskeleton-associated protein 4 (CKAP4) is significantly detectable in the blood serum of pancreatic tumor xenograft mice as well as patients diagnosed with PC. In contrast, CKAP4 is scarcely present in the serum of normal mice and postoperative patients. This confirms that CKAP4, a novel Dickkopf1 (DKK1) receptor secreted in exosomes, may serve as a biomarker for PC. Moreover, anti-CKAP4 monoclonal antibodies with different epitopes can inhibit the binding between DKK1 and CKAP4, AKT activity, as well as the proliferation and migration of PC cells. They can also suppress the formation of xenograft tumors in immunodeficient mice and extend the survival time of mice receiving intraperitoneal or orthotopic injection of PC cells. In conclusion, this CKAP4 targeting approach holds practical significance and is conducive to the future development of novel diagnostic and therapeutic methods for PC.

### Targeting exosome-secreted MiRNAs

MicroRNAs (miRNAs) are small non-coding single stranded RNAs approximately 22 nucleotides in length, which can regulate gene expression at the post transcriptional level. Recent studies have shown that they are involved in tumor evolution, including the regulation of angiogenesis and the development of therapeutic resistance [182]. As mentioned in gene therapy research, the role of miRNAs in the development of PC has become a hot topic in the scientific community. Through miRNA expression profiling analysis, researchers have revealed the abnormal expression of miRNAs in the serum and tumor tissues of PC patients. These abnormally expressed miRNAs are closely related to the disease stage, drug resistance, and survival of PC patients, thus having the potential to serve as biomarkers [183]. In summary, targeting these specific oncogenic miRNAs can provide an effective and optimal approach for the treatment of PC. In existing studies, miR- 193b-3p derived from M2 macrophage -derived exosomes enhances the proliferation, migration, invasion, and glutamine uptake of PC cells by targeting TRIM62, resulting in reduced ubiquitination of c-Myc. It has also been found that the expression of TRIM62 is negatively correlated with the expression of miR-193b-3p and c-Myc. High expression of miR-193b-3p and c -Myc predicts a poor prognosis for PC patients, while low expression of TRIM62 also indicates a poor prognosis for PC patients. Therefore, this represents a promising therapeutic target for PC and deserves continuous attention from researchers [184].

**Fig. 6:**
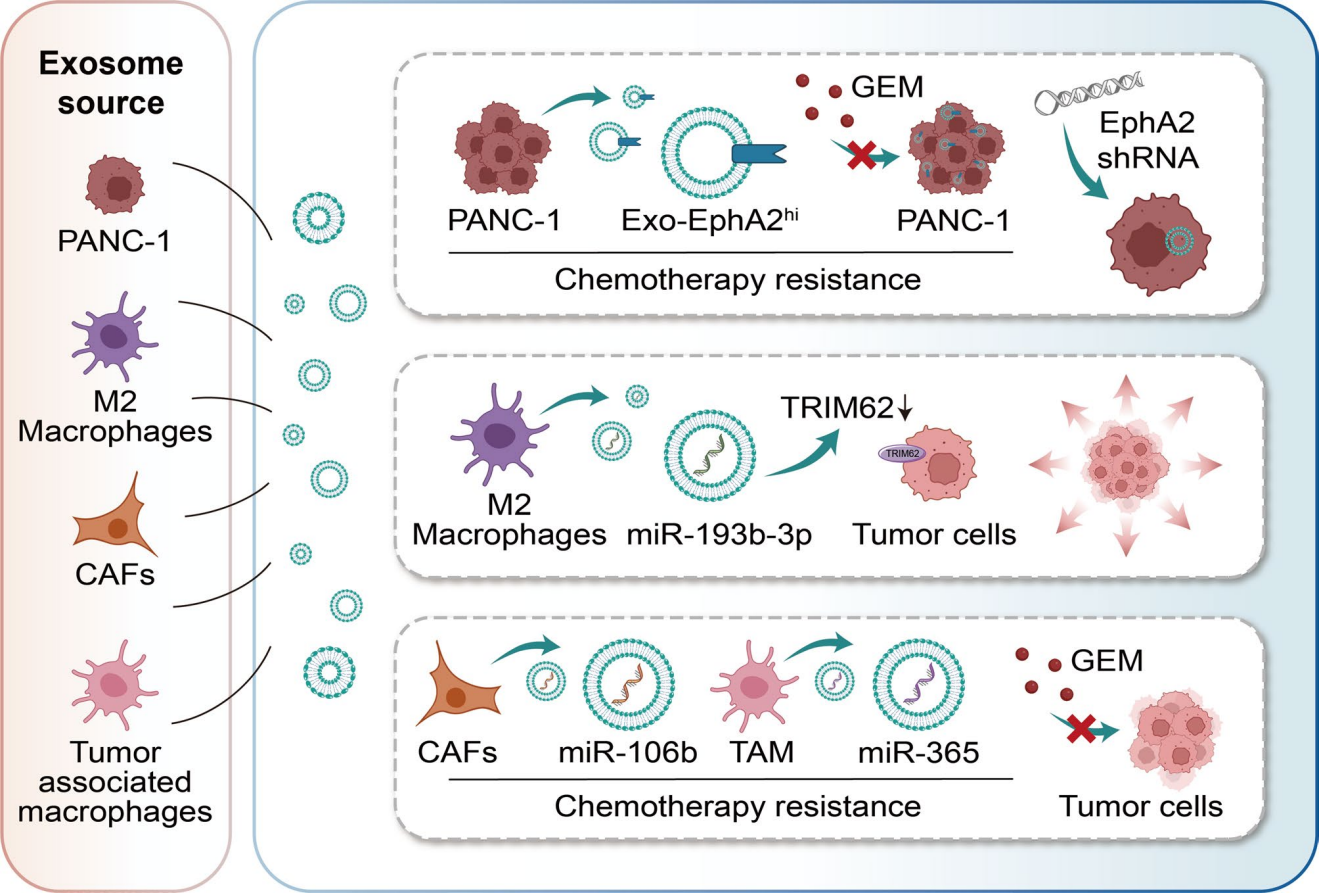
Applications of engineered exosome-based targeted strategies mainly include precision interventions involving: (1) Exosome-secreted protein biomarkers for PC. (2) Aberrantly expressed oncogenic exosome-secreted miRNAs. In this figure, we summarize some potential targets worthy of attention in future targeted strategies of PC

Besides their diagnostic significance, miRNA expression profiles might also help forecast resistance to chemotherapy (Fig. 6). GEM, which is a nucleoside analog of deoxycytidine, is frequently combined with other medications to treat different solid tumors. However, it has been shown in recent studies that various miRNAs can cause apoptosis evasion in PC cells, leading to increased resistance to GEM and poor prognosis of PC. Both in vitro and in vivo studies have broadly confirmed the predictive capability of miRNAs for GEM treatment response [185]. Exosomal miR-210 from PC stem cells encourages M2 macrophage polarization by targeting and suppressing FGFRL1, triggering the p-PI3K/p-AKT/p-mTOR signaling pathway, and enhancing resistance to GEM [186]. Similarly, miR-106b in cancer associated fibroblast (CAF) - derived exosomes [187] and miR-365 in tumor associated macrophage-derived exosomes [188] can reduce the sensitivity to GEM through their respective mechanisms. Using corresponding miRNA antagonists to counteract this effect has been proven to enhance the chemotherapy efficacy, which opens up a new avenue for the treatment of PC. As shown in Table 3, we summarize some representative applications of engineered exosome-based gene therapy and targeted therapy strategies in PC.

In summary, miRNAs from various sources hold significant potential as biomarkers for PC. However, they still face several challenges. Standardization in sample collection, isolation techniques, and data analysis is crucial for these endeavors [189]. It is hoped that future in-depth research on miRNAs will assist clinical practitioners in selecting a combination of therapies to overcome treatment resistance in PC.

## Clinical translation and challenges

PC remains one of the most challenging diseases to conquer in the medical field. Despite the need for more efforts and additional research, exosomes are gradually becoming a reality as targeted therapeutic drugs and drug delivery systems, not restricted to cancer. The progress in their development, along with advancements in molecular and nano engineering for precision medicine, will aid the scientific community in comprehending and creating improved treatment options in the future. Over the past few decades, scientists have conducted in-depth research on methods to extend the lifespan of exosomes. An example is phosphatidylserine, a significant phospholipid on exosome surfaces, which seems to serve as an ‘eat me’ signal in phagocytosis, boosting exosome clearance [190]. Thus, scientists including Kamerkar have illustrated that exosomes with CD47 on their surface send a regulatory signal that prevents them from being cleared by the mononuclear phagocyte system, resulting in prolonged circulation time [163]. However, there are challenges that need special consideration. Looking ahead, it is crucial to innovate more budget-friendly ways to produce extracellular vesicles and to implement safe cargo loading methods, which may include modifying the membrane. Furthermore, the deficiency of cutting-edge technologies for effective exosome purification drives the refinement of their isolation methods.Despite the widespread recognition of engineered exosome-based therapeutic strategies within the domain of PC pharmacotherapy, the majority of research remains preclinical, with numerous challenges confronting their practical application. Firstly, a major challenge in using exosomes as drug carriers is the absence of uniform methods for isolation and purification. Currently, there is no universally accepted protocol for obtaining clinical -grade exosomes, which limits their large -scale production and consistency [191]. Creating reliable and consistent methods for extracting exosomes is essential for their use in clinical environments [26].

Another challenge is the relatively poor efficiency in drug loading of exosomes. Common drug loading techniques, such as co-culture, sonication, and electroporation, each have their limitations. Co-culture is simple to operate but is only effective for hydrophobic molecules and has a low drug loading efficiency [78]. On the other hand, sonication and electroporation are more suitable for loading hydrophilic molecules and large biomolecules, but if not carefully controlled, these methods may also damage the exosome structure. Improving drug-loading efficiency without compromising exosome integrity remains a key research area. In addition, natural exosomes have limited targeting ability and are easily captured by macrophages in the body, which reduces their effectiveness as drug carriers. However, blocking the mononuclear phagocyte system can boost the delivery of engineered exosomes to the intended cells. By modifying exosomes to express molecules that resist phagocytosis, researchers are able to prolong their presence in the bloodstream, thereby achieving greater accumulation at the tumor site. Blocking the mononuclear phagocyte system can improve the delivery of engineered exosomes to target cells.

As part of cell bank characterization, exosome donor cells (e.g., MSCs or engineered lines) must be rigorously characterized—for cell identity, genetic stability, phenotype, morphology, viability, and the absence of contaminants such as mycoplasma, viruses, and endotoxins [192]. Furthermore, exosome culture conditions (media composition, oxygen/carbon dioxide levels, seeding density, and passage number) can impact exosome yield and cargo; therefore, process parameters must be tightly controlled to ensure reproducibility [193]. Currently, there are no universally accepted standards for physicochemical metrics: size distribution, particle count, protein content, potency, and purity thresholds must be defined and validated in-house. Batch-to-batch heterogeneity is a concern; comparability studies are crucial, especially after changes in the manufacturing process [194]. Exosomes must typically be stored at − 80 °C; however, the lack of validated shelf-life data and long-term stability information complicates logistics and regulatory approval [192]. The transition from bench-scale to GMP-grade, large-scale production requires advanced infrastructure, validated upstream and downstream processes, and a robust supply chain—all of which remain immature in this field [195].

Although exosome therapies have a low incidence of serious adverse events (approximately 0.7%) and an overall acceptable safety profile (approximately 4.4%), study heterogeneity masks subtle immunogenicity risks. The purity of the exosome loading is crucial: contaminating viral proteins, cytokines, or tumor-derived substances may pose safety concerns, especially when the donor cell source is not fully controlled [194].

Currently, there is no globally harmonized regulatory framework; classifications vary: the FDA considers exosomes to be biologics under Sect. 351, while the EMA may classify MSC-derived exosomes as active active pharmaceutical ingredients (ATMPs) [196]. The lack of standardized reporting metrics in exosome clinical trials limits cross-study comparisons. Harmonized guidelines are still under development. Inconsistent reporting of (serious) adverse events in early trials of exosome therapies has made safety analysis difficult [194].

## Conclusion and future prospects

Exosomes, as a natural delivery system, offer distinct benefits like minimal toxicity, targeting ability, and immune-modulating characteristics, making them ideal for cancer treatment. They can precisely deliver chemotherapeutic agents, antigens, antigen-presenting molecules, and immunomodulatory molecules to tumors, overcoming obstacles like low immunogenicity and immunosuppressive microenvironments. Therapeutic strategies based on engineered exosomes, including increasing antigen release, expression, and presentation, reprogramming tumor-associated macrophages, targeting regulatory T cells and myeloid-derived suppressor cells, along with bettering the physical and chemical attributes of the TME, offer fresh potential for improving PC therapy. Our team believes that although engineered exosomes are still in the preclinical stage, they have laid the foundation for clinical translation. The next steps should focus on GLP toxicology studies, pharmacokinetic assessments, and dose-escalation experiments. The drug delivery system of engineered exosomes has great potential in PC treatment, and exploring various combination therapies based on this system is key to enhancing efficacy and overcoming resistance. With ongoing advancements in new technologies and innovative, multidisciplinary approaches to clinical translation, it is believed that engineered exosome therapy could become a frontier in cancer treatment.

**Table 1:** Summary of applications of engineered exosome-based chemotherapy strategies in PC

Exosome source	Valid cargo	Model type	Therapeutic effect	Ref.
M1 macrophages	GEM, Deferoxamine	GEM-resistant PANC−1/GEM cell line	Restrict the formation and proliferation of PANC−1/GEM tumor spheroids	[92]
BM-MSCs	PTX, GEM monophosphate	PDAC orthotopic model	Demonstrate enhanced homing and penetration abilities while maintaining low systemic toxicity	[94]
PANC−1	GEM	Tumor-bearing mice	ExoGEM treatment led to the disappearance of tumors in several mice, with no recurrence	[95]
MSCs	PTX	Murine SR4987 line/human PC line CFPAC−1	Demonstrate a strong anti proliferative activity on CFPAC−1	[103]
Gingival MSCs	PTX	Human PC line CFPAC−1	Exert a significant anticancer effect	[104]
PANC−1	PTX, RGD magnetic nanoparticles	H729/PANC−1 xenograft mice	Show a significant reduction in tumor size	[105]
PC cells, pancreatic stellate cells, macrophages	DOX	PC cell lines	exosomes have donor - cell - specific differences, which may affect their therapeutic effects as drug carriers	[110]
THP−1 cells	DOX, magnetic nanoparticles	Tumor-bearing mice	Obvious anti-tumor and tumor-reducing effects	[111]

**Table 2:** **S**ummary of applications of engineered exosome-based immunotherapy strategies in PC

Exosome source	Valid cargo	Model type	Therapeutic effect	Ref.
Adipose-derived stem cells	PD-L1 siRNA, CD−133-binding peptides	Murine models with ASPC−1 cells	Achieve significant anti-cancer effects by specifically attaching to CD133-positive PC cells and reducing the expression of PD-L1	[118]
Tumor cells	Exo70 inhibitor	Tumor-bearing mice	Reduce the release of tumor exosomes and PD-L1 expression, inhibiting immune evasion	[119]
BM-MSCs	Kras G12D specific siRNA	Advanced PDAC samples	Downregulation of KRAS G12D DNA and suppression of phopho-Erk	[121]
BM-MSCs	Galectin−9 siRNA Oxaliplatin prodrug	PC cell lines, C57BL/6 mice	Enhance tumor targeting, improving the immunogenic cell death response and trigger an anti-tumor immune response	[122]
Immunogenically dying tumor cells	MART−1 peptide CCL22 siRNA	Tumor-bearing mice	inhibit tumor progression in both prophylactic and combination chemotherapy treatment models	[132]
PANC−1	Ultrafiltered exosome lysates	PANC−1 cells	DC/CIKs stimulated by UEL demonstrated a higher killing rate compared to those stimulated by LPS and PE	[142]
Tumor cells	DC cells, GEM, ATRA, Sunitinib	UNKC6141 PaCa line	Act at different stages of maturation and activation of MDSCs, prolong the survival of mice	[144]
Tumor cells	Photosensitizer chlorophyll e6, laser irradiation	Tumor-bearing mice	Generate reactive oxygen species efficiently in tumor cells and boost cytokine secretion from immune cells	[145]

**Table 3:** Summary of applications of engineered exosome-based gene therapy and targeted therapy strategies in PC

Exosome source	Valid cargo	Model type	Therapeutic effect	Ref.
Tumor cells	SiPAK4	PC tumor mouse model	Minimize PC tumor growth in living organisms and improve mouse survival with low toxicity	[147]
Tumor cells	GW4869	PC cell lines	The presence of GW4869 can reverse the inhibited response to treatment	[160]
Periodontal ligament stem cells	GEM, a suicide gene fusion of yeast cytosine deaminase	PC cell lines	Impose further inhibition on cancer cell growth, thus hindering the proliferation of PC cells	[161]
Umbilical cord MSCs	MiR-145-5p	PC tumour mouse model	Limit PDAC cell growth and invasion, increase apoptosis and cell cycle arrest, while Smad3 expression is decreased in vitro	[166]
Stroma	MiRNAs derived from the tumor-associated stroma	Co-culture of primary human pancreatic TAS cells with xenograft PDAC cells	Through exosomes, TAS-derived miRNAs reach adjacent PDAC cells and reduce tumor cell growth	[167]
HEK293 cells	MiR−34a	PC cell lines and xenograft nude mice models that carry Panc28 cells	Prevent the proliferation of PC both in vitro and in vivo	[168]
MSCs	CRISPR/Cas9 plasmid DNA	Subcutaneous and orthotopic models of PC	Direct efforts towards the mutant Kras G12D oncogenic allele in PC cells to curb proliferation and halt tumor expansion	[171]
PC cell lines: PANC−1, MIA PaCa−2, and BxPC−3	EphA2-knockdown	A chemoresistant PC cell line treated with or without EphA2 shRNA	Reduce EphA2 expression in PANC−1 cells hindered their capacity to convey exosome-mediated chemoresistance to MIA PaCa−2 and BxPC−3	[180]
M2 macrophages	MiR−193b−3p inhibitor	SW1990 cells	The proliferation, migration, invasion, and glutamine uptake of SW1990 cells were stimulated by miR−193b−3p	[184]
CAFs	MiR−106b inhibitor	PC cell lines	Lowering miR−106b levels in CAFs-exosomes leads to reduced resistance of cancer cells to GEM	[187]
Tumor associated macrophages	MiR−365 antagonist	Genetic mouse model of PDAC	Immune transfer of the miR−365 antagonist recovered the sensitivity to gemcitabiner	[188]

## Data Availability

No datasets were generated or analysed during the current study.

## Abbreviations

5-FC: 5-fluorocytosine
5-FU: 5-fluorouracil
ACT: Adoptive cell therapy
ATRA: All-trans retinoic acid
CAF: Cancer associated fibroblast
CAR: Chimeric antigen receptor
Ce6-R-Exo: Chlorin e6 photosensitizer-modified tumor-derived recombinant exosomes
CIK: Cytokine - induced killer
CKAP4: Cytoskeleton-associated protein 4
CTLA-4: Cytotoxic T lymphocyte-associated antigen 4
DC: Dendritic cell
DC-TEX: DC vaccines loaded with tumor-derived exosomes
DKK1: Dickkopf1
DOX: Doxorubicin
ECM: Extracellular matrix
EMT: Epithelial-mesenchymal transition
EphA2: Ephrin type-A receptor 2
GEM: Gemcitabine
HUVECs: Human umbilical vein endothelial cells
ICIs: Immune checkpoint inhibitors
miRNAs: MicroRNAs
MSC-exosomes: Mesenchymal stem cell-derived exosomes
MSCs: Mesenchymal stem cells
ncRNAs: Non-coding RNAs
NK: Natural killer
PAK4: P21 - activated kinase 4
PC: Pancreatic cancer
PD-1: Programmed death 1
PD-L1: Programmed death-ligand 1
PTX: Paclitaxel
R-Exo: Recombinant exosomes
RGD: Arginine-Glycine-Aspartic Acid
RNAi: RNA interference
shRNAs: Short hairpin RNAs
siRNAs: Specific small interfering RNAs
TAAs: Tumor associated antigens
TAS: Tumor-associated stroma
TCR: T cell receptor
TEXs: Tumor-derived exosomes
TIL: Tumor-infiltrating lymphocyte
TME: Tumor microenvironment
TRAIL: Tumor necrosis factor-related apoptosis-inducing ligand
TSAs: Tumor- specific antigens
VEGF: Vascular endothelial growth factor
